# Sex-related difference of subcortical gray matter volume associated with crystallized intelligence in young adults with obesity

**DOI:** 10.1186/s13293-026-00850-8

**Published:** 2026-02-08

**Authors:** Junhao He, Guanjinghui Xu, Junjie Zhao, Yang Wang, Chenxin Lou, Siqi Wu, Haocheng Zhan, Hui Xu

**Affiliations:** 1 School of Mental Health, Wenzhou Medical University, Wenzhou, 325035 China; 2 School of the 1st Clinical Medical Sciences, School of Information and Engineering, Wenzhou Medical University, Wenzhou, 325035 China; 3 The Affiliated Kangning Hospital of Wenzhou Medical University, Zhejiang Provincial Clinical Research Center for Mental Health, Wenzhou, 325007 China; 4 Key Research Center of Philosophy and Social Sciences of Zhejiang Province, Institute of Medical Humanities, Wenzhou Medical University, Wenzhou, 325035 China

**Keywords:** Obesity, Cortical thickness, Cortical surface area, Right amygdala, Gray matter volume, Crystallized intelligence

## Abstract

**Background:**

Overwhelming evidence shows that obesity is associated with brain structural alterations in young adults. However, the specific sex related changes in obesity and their associated with crystallized intelligence still remained unclear.

**Methods:**

In this study, 243 young adults with obesity and matched 243 lean individuals were enrolled from the Human Connectome Project Release S1200 dataset. Surface-based morphometry approach was adopted to investigate altered brain structures and related sex difference in young adults with obesity with three structural indicators including cortical thickness (CT), cortical surface area (CSA), and subcortical gray matter volume (GMV).

**Results:**

While in female young adults, no significant difference of GMV of right amygdala was observed between obesity and lean groups, in male young adults, obesity group exhibited significantly higher GVM of right amygdala than lean group. Then, obesity male young adults showed significant correlation between GMV of right amygdala and crystallized intelligence, and such significant correlation was not found in obesity female young adults.

**Conclusions:**

Obesity male young adults showed significantly higher GMV of right amygdala than lean male young adults, which was further associated with crystallized intelligence. Such findings were not observed in obesity female young adults. These findings suggested that the sex difference of adverse effect of obesity may be associated with the alterations of GMV of right amygdala.

**Supplementary Information:**

The online version contains supplementary material available at 10.1186/s13293-026-00850-8.

## Highlights

- Obesity male young adults exhibited significantly higher GVM of right amygdala than lean group.
- Obesity male young adults showed significant correlation between GMV of right amygdala and crystallized intelligence.
- Such findings were not observed in obesity female young adults.

**Supplementary Information:**

The online version contains supplementary material available at 10.1186/s13293-026-00850-8.

## Introduction

Obesity, defined as excessive fat accumulation that adversely impacts health, has evolved into a global pandemic [1]. According to the 2024 report of World Health Organization, the age-standardized prevalence of obesity with body mass index (BMI) ≥ 30 kg/m² among adults aged 18 years and older has risen eightfold since 1990, reaching 8.0% in 2022. Globally, the combined prevalence of overweight and obesity increased from 21.1% in 1990 to 23.0% in 2022, driven predominantly by the surge in obesity, which more than doubled from 6.6% to 15.8% over the same period. Notably, sex disparities persist, that women exhibiting a consistently higher obesity prevalence than men. Additionally, in 2022, the age-standardized obesity rate was 17.9% in women, exceeding that of men by 4.3% points [2, 3].

From a physiological perspective, obesity is increasingly recognized as a manifestation of neuropsychological disorders [4]. Although discussions about obesity often focus on its physiological effects, the potential impact on other health issues (e.g., mental health) is frequently overlooked. Most evidence indicates that central inflammation induced by obesity is associated with adverse cognitive outcomes [5]. A growing body of evidence further links obesity to various cognitive dysfunctions, including impaired decision-making [6], deficits in inhibitory control [7], learning/memory impairments [8], and attentional problems [9]. Adolescents with obesity exhibit significantly lower health-related quality of life compared to their normal-weight peers [10].Neurobiological studies have revealed that obesity was associated with brain structural and functional alterations, including lower cortical thickness (CT) ofthe hypothalamus and orbitofrontal cortex, which were further linked to deficits in fluid reasoning, a core aspect of cognitive function [11]. Furthermore, obesity-related demyelination and oligodendrocyte dysfunction contribute to cognitive and motor deficits, as evidenced by impaired executive function and poor working memory [12, 13]. Meanwhile, weight loss may partially reverse neurostructural degeneration [14]. Previous studies have demonstrated that greater weight loss correlates with higher CT and greater cognitive performance [15, 16]. Additionally, obesity might be associated with psychiatric disorders, as evidenced by the positive causal relationship between higher cortical surface area (CSA) in the transverse temporal lobe among individuals with high-BMI and elevated risks of attention-deficit/hyperactivity disorder and Alzheimer’s disease [17, 18].

Furthermore, obesity is associated with an increased risk of various adverse psychiatric disorders, with reported psychiatric disorder risks ranging from 30% to 70% among individuals with obesity [19]. Obesity is linked to multiple psychiatric conditions, including lifetime mood disorders, anxiety, personality disorders, attention-deficit/hyperactivity disorder (ADHD), binge-eating disorder, trauma, and schizophrenia [20]. Most relevant studies demonstrate that higher BMI correlates with poorer mental health, particularly depression and subclinical depressive symptoms [21]. Notably, in overweight or children and adolescents with obesity, elevated BMI is associated with increased risk of anxiety and depression [22]. Patients seeking weight loss treatment have a higher prevalence of ADHD (26–61%), suggesting an association between ADHD and obesity [23]. Compared with the general population, the prevalence of obesity is nearly twice as high in patients with major depressive disorder and bipolar disorder [24]. Overall, the incidence of mental health disorders is significantly higher in individuals with obesity versus their normal-weight peers [25, 26]. In severe cases, it may progress to autism spectrum disorder [14]. Obesity often co-occurs with mental disorders at a high prevalence rate [27].

Critically, sex differences further complicate the impact of obesity. Young men appear to have a lower risk of obesity and psychiatric disorders than young women [20]. Women, individuals with obesity, and those with preexisting mental health conditions seem particularly vulnerable to emotional eating triggered by stress [28]. Overweight or children and adolescents with obesity are more likely to develop depression or exhibit more depressive symptoms than their normal-weight peers, and this association is more pronounced in females [29]. The onset of post-traumatic stress disorder (PTSD) symptoms is associated with an increased risk of overweight or obesity [30, 31]. Furthermore, it is now found that men with obesity have a lower prevalence of PTSD related to obesity development than women with obesity [20]. Obesity significantly increases the odds of developing various psychiatric disorders across all age groups, including depression, psychotic spectrum disorders, anxiety disorders, schizoaffective disorder, eating disorders, and personality disorders. Additionally, studies have found significant gender differences in most of these disorders: except for schizophrenia and nicotine addiction, men have a lower risk of developing all these disorders than women [32].A notable feature of sex-specific brain structural changes in obesity is the lower gray matter volume (GMV) in the nucleus accumbens, orbitofrontal cortex, and globus pallidus in females, coupled with downregulation of D2/D3 receptors in the striatum [33]. This may explain why female with obesity, but not male, exhibit a stronger preference for immediate rewards despite long-term negative consequences [34, 35].

Furthermore, neuroimaging studies reveal that females with high BMI demonstrate greater centrality in reward network(amygdala, hippocampus, and nucleus accumbens) and salience network (anterior mid-cingulate cortex), whereas males exhibit greater centrality in reward network (putamen) and sensorimotor network (posterior insula). These findings suggest that obesity is associated with sex-dependent topological restructuring in reward, salience, and sensorimotor networks [36]. However, there were still several limitations among previous research. Firstly, small sample size with less than 100 was adopted in previous studies, which would affect statistical power and finding reliability [18]. Secondly, previous studies investigating abnormal brain structures in obesity conducted only one structural indicator, such as CT or CSA or GMV [16]. Thirdly, the sex-specific neurobiological mechanism about subcortical changes underlying young adults with obesity was not fully explored [7].

To address these gaps, this study adopted surface-based morphometry to investigate altered brain structures and examined related sex difference in young adults with obesity with three structural indicators including CT, CSA, and GMV together. Specifically, this study aimed to (1) assess sex related difference in CT, CSA and GMV in young adults with obesity; and (2) explore the relationship between sex related difference of brain structures with cognitive function in young adults with obesity. We hypothesized that (1) young adults with obesity exhibited sex-related difference of subcortical GMV, and (2) sex-related difference of subcortical GMV associated with intelligence ability in young adults with obesity.

## Methods

### Participants

All participants in this study were enrolled from the Human Connectome Project (HCP) Release S1200 dataset [37]. All participants provided written informed consent and were young adults aged 22–35 years. The exclusion criteria were as follows: history of psychiatric disorder, substance abuse, neurodevelopmental disorder or damage, cardiovascular disease, severe health conditions (diabetes, multiple sclerosis, cerebral palsy, and premature birth), or MRI contraindications. The complete details of the inclusion and exclusion criteria and informed consent for all participants can be found in previous research [37, 38]. In this study, all participants were categorized into two groups according to their BMI (young adults with obesity: BMI > 30; lean individuals: 18 < BMI < 25) [39, 40]. Individuals with a BMI between 25 and 30 were excluded to create two separate groups of individuals with clinically significant differences in BMI. Furthermore, the R-based “MatchIt” package was adopted to match young adults with obesity and lean individuals in terms of demographic variables including sex, age, total family income and education level. Hence, the total sample in this study comprised 486 participants (243 young adults with obesity, and 243 lean individuals, Table 1). The MatchIt provides a simple and straightforward interface to various methods of matching for covariate balance, which has been adopted in previous studies [41, 42]. This study was approved by the research ethics board of each institution and was conducted in accordance with the Declaration of Helsinki.

**Table 1:** Demographic and cognitive assessments in obesity group and lean group

	Obesity group		Lean group		Group-by-Sex interaction effect	
	Female (*N* = 140)	Male (*N* = 103)	Female (*N* = 131)	Male (*N* = 112)	F-value	*p*-value
Age (years)	30.100(3.549)	28.204(3.658)	29.656(3.622)	27.964(3.609)	0.096	0.757
Total family income	4.414(1.967)	4.854(2.017)	4.626(2.157)	4.000(2.110)	7.991	0.005
Education level	14.250(1.979)	14.641(1.770)	14.595(1.880)	14.536(1.739)	1.764	0.185
Sleep quality	5.779(3.107)	4.864(2.540)	4.809(2.754)	4.768(2.543)	2.969	0.086
Impulsive trait	0.335(0.222)	0.335(0.202)	0.355(0.202)	0.411(0.255)	1.897	0.169
Fluid cognitive ability	100.450(15.494)	105.800(19.187)	103.691(15.038)	104.474(17.146)	2.231	0.136
Crystal cognitive ability	103.292(19.921)	113.123(21.926)	109.111(19.615)	113.804(18.943)	1.935	0.165
General cognitive ability	101.182(17.090)	108.646(17.571)	106.523(16.647)	112.021(16.469)	0.400	0.528
WM accuracy	83.076(9.200)	86.732(9.356)	85.592(9.132)	89.136(7.830)	0.005	0.946
WM RT	871.395(130.715)	845.021(110.732)	873.203(124.575)	870.515(144.580)	0.976	0.324
2Back-WM accuracy	78.756(10.874)	83.738(10.942)	81.801(11.326)	85.667(10.268)	0.304	0.582
2Back-WM RT	969.563(148.399)	953.149(132.661)	975.086(145.523)	976.746(159.410)	0.435	0.510
0Back-WM accuracy	87.063(11.174)	89.805(10.911)	89.070(10.376)	92.339(8.823)	0.075	0.784
0Back-WM RT	780.595(140.629)	744.461(115.238)	778.819(135.125)	772.646(151.268)	1.382	0.240
Aggressive behavior level	53.094(5.256)	53.686(4.957)	52.374(3.676)	52.839(3.864)	0.024	0.877
Total problems level	48.417(9.131)	49.647(8.369)	47.344(9.818)	49.143(9.092)	0.115	0.734

All data was presented as mean (standard deviation) unless otherwise stated. *P*-adjusted: *P* value after false discovery rate correction; Sleep quality was indexed by Pittsburgh Sleep Quality Index total score; Impulsive trait was indexed by the average of Delay Discounting Area Under the Curve for both $200 and $400; WM: working memory; RT: response time; Aggressive behavior level was indexed by adult self-report aggressive behavior score; Total problems level was indexed by adult self-report total problems score

### Clinical and neuropsychological assessments

For clinical assessments, sleep quality was indexed using the total score from the Pittsburgh Sleep Quality Index (PSQI) [43], and impulsive trait was measured as the average of the delay discounting area under the curve for both $200 and $40K. Based on Green and Myerson’s method, six level of fixed delays (1 month, 6 months, 1 year, 3 years, 5 years, and 10 years) and two level of adjusted rewards ($200 and $40K) were employed to determine indifference points, with AUC serving as a robust measure of individuals’ impulsivity [44, 45]. Furthermore, their aggressive behavior level and total problems level was indexed by adult self-report aggressive behavior score and adult self- report total problems score respectively [46].

Neurocognitive ability was examined across several cognitive domains, including general cognitive ability and working memory [47]. Fluid intelligence was calculated by averaging the normalized scores from each of the toolbox tests that measure fluid abilities, including the Flanker, Dimensional Change Card Sort, Picture Sequence Memory, List Sorting, and Pattern Comparison [48]. Similarly, the crystallized intelligence was derived by averaging the normalized scores of each of the Toolbox tests that are crystallized measures, including Picture Vocabulary and Reading Tests [49]. Hence, the general cognitive ability was derived by averaging the scores of the fluid cognitive ability and crystallized cognitive ability. Higher scores indicate higher levels of cognitive ability. Working memory was measured using the N-Back paradigm, which includes assessments of accuracy and response time (RT) for both 0-back and 2-back tasks. All Clinical and neuropsychological assessments were summarized in Table S1.

### MRI data acquisition

All participants underwent MRI scans using a 3.0T MRI scanner. A custom-built head holder was employed to secure participants’ heads during scanning. Standard T1-weighted anatomical data were acquired using a three-dimensional magnetization-prepared rapid gradient echo sequence (echo time = 3.17 ms, repetition time = 8.15 ms, flip angle = 9°, slice thickness = 1 mm, field of view = 256 mm × 256 mm, matrix size = 256 × 256, acquisition time = 4 min, 30 s). During the scan, participants were instructed to keep their eyes closed and refrain from engaging in cognitive or motor-related activities. The alertness of the participants was confirmed immediately after the scan.

### MRI data preprocessing and measurement of CT, CSA and GMV

Structural T1-weighted MRI data for each participant were processed using the FreeSurfer software package (http://surfer.nmr.mgh.harvard.edu). More details on surface-based morphology analysis have been provided in previous studies [50]. In summary, the FreeSurfer pipeline encompassed the following steps: removal of non-brain tissue, automated Talairach transformation of each participant’s native brain, intensity normalization, tessellation of the gray/white matter boundary, automated topology correction, surface deformation following intensity gradients, registration of the participant’s native brain to a common spherical atlas, and reconstruction of the cortical surface. To obtain CT and CSA measurements, the cortical morphologies were smoothed using a 10 mm full-width-at-half-maximum Gaussian kernel, a method previously employed in previous research [51]. This smoothing process was repeated using the same kernel size to ensure accurate CSA and CT measurements. During preprocessing, all outputs were subject to meticulous accuracy inspection, with manual corrections applied where necessary. Subsequently, the average CSA and CT value within the 34 cortical regions, which were defined by the Desikan atlas [52] in each hemisphere, was determined. And GMV for subcortical regions from the ASEG parcellation plus the intracranial volume (ICV) were derived in FreeSurfer.

### Statistical analysis

Demographic, clinical characteristics and sMRI variables of all participants were analyzed using R (Version 4.1.3; R Core Team, 2022) and RStudio (“Ghost Orchid” Release; RStudio Team, 2021). For demographic, clinical characteristics and ICV, two-way ANOVA with the first between-subjects factor “group” (obesity and lean) and the second between-subjects factor " sex " (male and female) were performed. When the group-by-sex interaction was significant, simple effect analysis was performed, and p values were false discovery rate (FDR) corrected. Then, any above variables showed significant interaction effect were included as covariates in the following analysis of sMRI variables.

For sMRI variables, linear mixed effect (LME) model was conducted for each sMRI variable. Total family income and ICV showed significant interaction effects were regarded as fixed effects. A threshold of *p* < 0.05 was used to indicate significance.

Then, correlation analysis was conducted between clinical characteristics and sMRI variables which exhibited significant interaction effect in lean group and obesity group respectively. The significance threshold was set at *p* < 0.05 after FDR corrected. Then, correlation analysis was further performed to examine whether similar relationship can be found in both male young adults and female young adults.

## Results

### Participants and characteristics

**Fig. 1:**
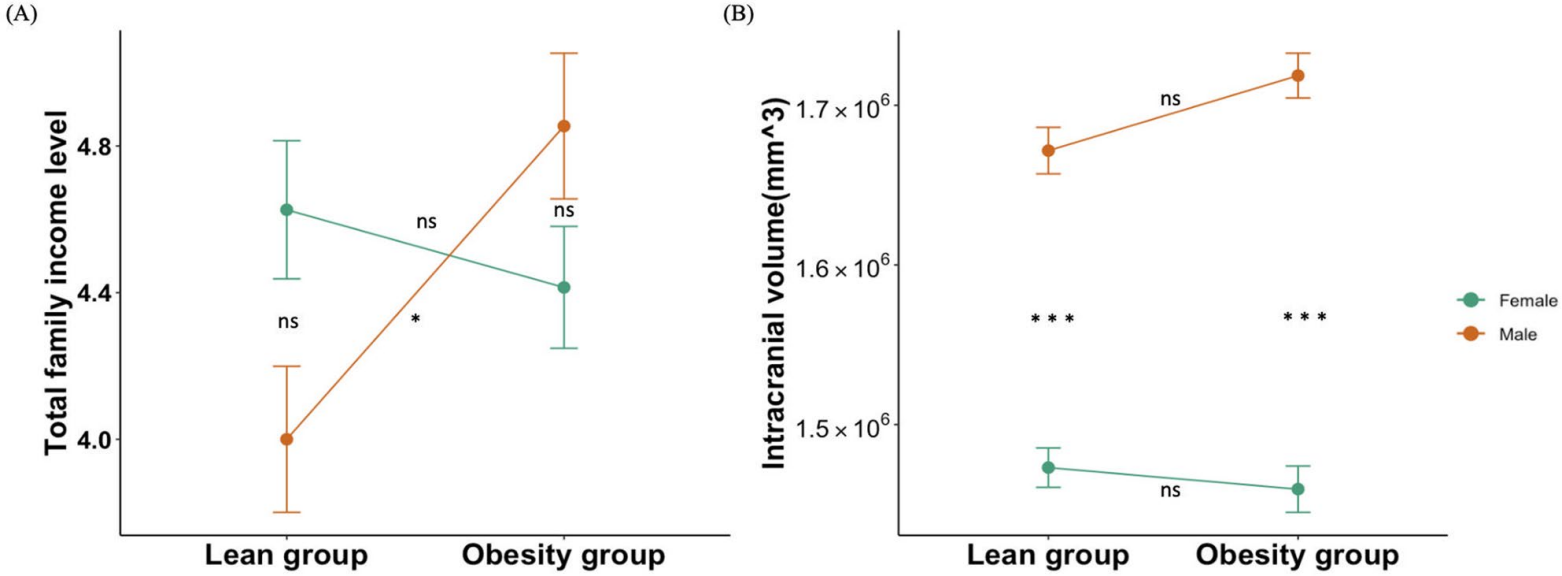
Significant “group” $\:\times\:$ “sex” interaction effect on (**A**) total family income and (**B**) intracranial volume. The red line represented male young adults, and the green line represented female young adults. ns, p $\:>$ 0.05; *, p $\:<$ 0.05; ***, p $\:<$ 0.001

There was significant “group” $\:\times\:$ “sex” interaction effect on total family income (F $\:=$ 7.991, p $\:=$ 0.005, Fig. 1A). Further simple effect analysis found that in male young adults, obesity group showed significant higher total family income level than lean group (p $\:=$ 0.014), and no sigficant group difference was observed in female young adults (p $\:=$ 0.833). Additionally, there was significant “group” $\:\times\:$ “sex” interaction effect on ICV (F $\:=$ 7.991, p $\:=$ 0.005, Fig. 1B). Further simple effect analysis found that in both lean and obesity group, male young adults showed significantly higher ICV than female young adults (p $\:<$ 0.001).However, there were no significant “group” $\:\times\:$ “sex” interaction effect on other demographic and cognitive assessments variables (p $\:>$ 0.05).

### Sex-related difference of subcortical GMV

**Fig. 2:**
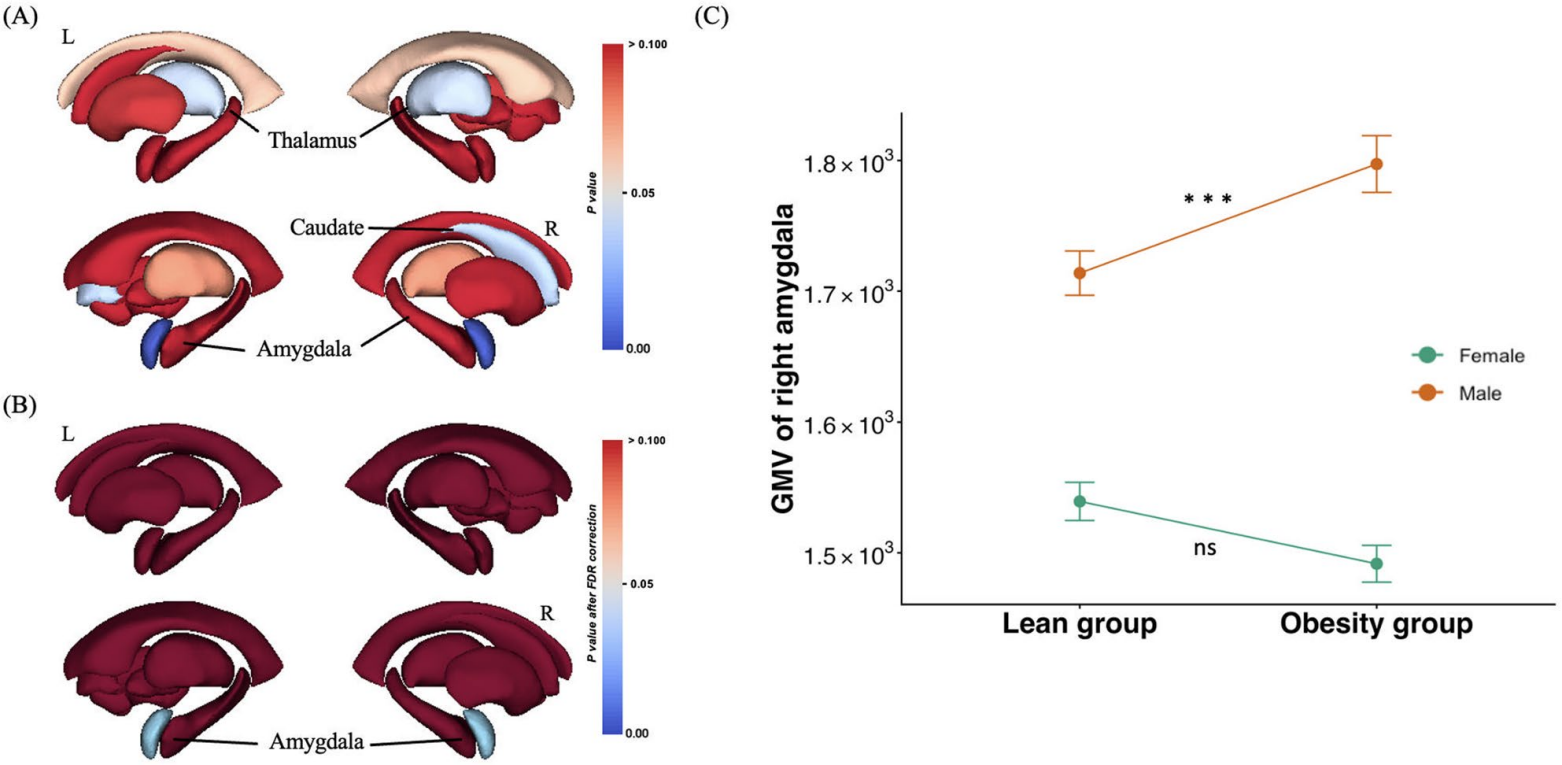
(**A**) significant “group” $\:\times\:$ “sex” interaction effect for left thalamus, right caudate, and right amygdala with uncorrected p. The color bar represented uncorrected p value. (**B**) after FDR correction, there was only significant “group” $\:\times\:$ “sex” interaction effect observed in right amygdala. The color bar represented p value after FDR correction. (**C**) Simple effect analysis for right amygdala. The red line represented male young adults, and the green line represented female young adults. ***, p $\:<$ 0.001

In terms of subcortical GMV, left thalamus, right caudate, and right amygdala showed significant “group” $\:\times\:$ “sex” interaction effect (F $\:=$ 2.043, uncorrected p $\:=$ 0.042; F $\:=$ 2.079, uncorrected p $\:=$ 0.038; F $\:=$ 3.172, uncorrected p $\:=$ 0.001 respectively, Fig. 2A). However, after FDR correction, there was only significant “group” $\:\times\:$ “sex” interaction effect observed in right amygdala (corrected p $\:=$ 0.029, Fig. 2B), and further simple effect analysis found that in female young adults, no significant difference of GMV of right amygdala was observed between obesity and lean groups, but in male young adults, obesity group exhibited significantly higher GVM of right amygdala than lean group (p $\:<$ 0.001, Fig. 2C).

Additionally, LME models were conducted on both CT and CSA, and there were no significant “group” $\:\times\:$ “sex” interaction effect on all cortical regions based on CT (Figure S1) and CSA (Figure S2).

### Correlation analysis

**Fig. 3:**
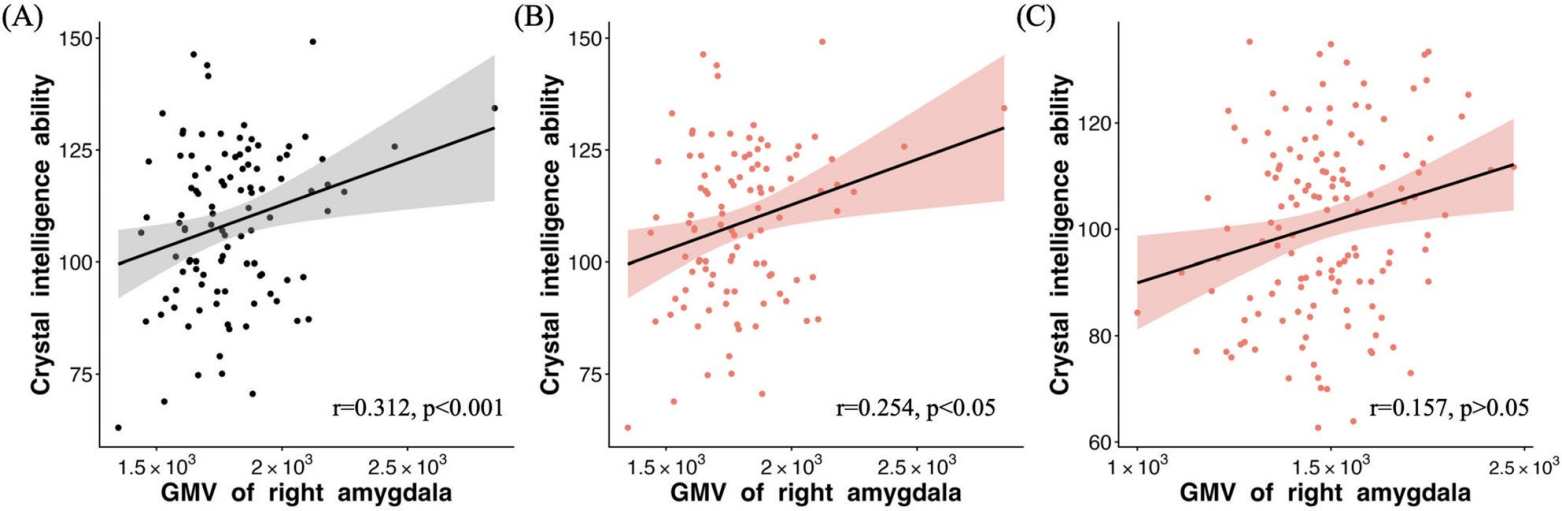
(**A**) Significant correlation between GMV of right amygdala and crystallized intelligence was found in obsity group; (**B**) Significant correlation between GMV of right amygdala and crystallized intelligence was observed in obesity male young adults; (**C**) no significant correlation was found between GMV of right amygdala and crystallized intelligence in obesity female young adults. GMV, gray matter volume

In lean group, there were no significant correlations between GMV of right amygdala and fluid intelligence or crystallized intelligence (*p* > 0.05 after FDR correction). In obesity group, while no significant correlation was observed between GMV of right amygdala and fluid intelligence (*p* > 0.05 after FDR correction), there was significant correlation found between GMV of right amygdala and crystallized intelligence (*p* < 0.05 after FDR correction, Fig. 3A).

Then, further correlation analysis was performed to examine whether similar relationship can be found in both male young adults and female young adults in obesity group. We found that there was significant correlation observed between GMV of right amygdala and crystallized intelligence in obesity male young adults (Fig. 3B), and no significant correlation was found between GMV of right amygdala and crystallized intelligence in obesity female young adults (Fig. 3C).

## Discussion

This study adopted surface-based morphometry approach to investigate altered brain structures and related sex difference in young adults with obesity with three structural indictors including CT, CSA, and subcortical GMV together. We found no significant differences in CT or CSA between the lean and obesity groups in young adults, which deviated from our expected findings. In previous literature, we noted that in children and adolescents, individuals with obesity showed lower cortical thickness [53], and CSA also exhibited varying alterations [54]. This was associated with reduced executive function (e.g., working memory) in children and adolescents with obesity [53]. However, in middle-aged and older adults, sex differences emerged: middle-aged women over 50 years old had lower CT compared to men [55]. We found that in both lean and obesity group, male young adults showed significantly higher GMV of right amygdala than female young adults. Additionally, in the male group, obesity group showed significantly higher GMV of right amygdala than lean group. In obesity group, significant correlation between GMV of right amygdala and crystallized intelligence in all young adults, as well as in male young adults and female young adults. These findings throw light on sex-related difference of subcortical GMV associated with intelligence in young adults with obesity.

Consistent with previous research, our findings revealed that male young adults in both the lean and obesity groups exhibited significantly higher ICV than female young adults. Previous research highlights that male brains are larger than female brains from birth, with this size difference stabilizing at approximately 11% in adulthood. This divergence explains other reproducible outcomes, such as higher white-to-gray matter ratios, distinct intra- and inter-hemispheric connectivity patterns, and variations in regional cortical and subcortical volumes in males [56]. Our finding strongly supports significant sex-based differences in brain volume, confirming that males exhibit markedly larger ICV compared to females. Concurrently, another key finding reveals a within-group effect of overall family income on males in both the lean and obesity groups. Specifically, males in the obesity group demonstrated higher household income levels than those in the lean group. This finding further demonstrated that male participants in the obesity group were associated with elevated household income levels, consistent with broader research suggesting a positive correlation between national obesity prevalence and income levels. Global obesity rates are projected to rise continuously from 2019 to 2024 [57], a trend potentially linked to nutrition transitions marked by diets high in fats, sugars, and refined carbohydrates, alongside increasingly sedentary lifestyles [58]. Notably, sex disparities in obesity prevalence are evident: males exhibit higher obesity rates than females in most countries [59], and higher-income males are more susceptible to obesity than females [60]. This pattern may be driven by sex-specific social behaviors. Additional studies indicate that males have a 100% increased likelihood of obesity if a close male friend becomes obesity, whereas this social contagion effect is negligible among females [61].

Further, our study found that in both lean and obesity group, male young adults showed significant higher GMV of right amygdala than female young adults, and in male group, obesity group showed significant higher GMV of right amygdala than lean group, which was consistent with recent research increasingly underscoring the amygdala’s role in modulating obesity-related behaviors. Obesity-related activation of the amygdala by food cues has been documented in individuals with obesity [62]. Our study identified a correlation between obesity and GMV of right amygdala, aligning with findings by Opel et al. [63]. Specifically, males in the obesity group exhibit significantly larger right amygdala volumes compared to the lean group, corroborating previous research [64], which reported reduced lateralization indices and right amygdala enlargement in populations with obesity. This phenomenon may involve obesity-induced neuroinflammation and sex hormone interactions. Adipose tissue inflammation, driven by dysfunctional adipocytes and immune cell infiltration [65], has been shown to affect brain structures such as the hippocampus and amygdala. Inflammatory cytokines are implicated in accelerating amygdala volume expansion [5, 66]. Additionally, a sex-specific interaction emerged: obesity positively influenced GMV of amygdala in males but had no significant effect in females. This divergence may stem from gonadal hormone regulation. Further animal studies indicate that the amygdala is a key target for gonadal hormones, with dense distributions of androgen and estrogen receptors in this region [67, 68]. Estrogens, such as estrogen, have been associated with reduced GMV of right amygdala [69, 70], whereas androgens lack analogous effects. The antagonistic interplay between gonadal hormones and obesity-related neuroinflammation may explain the absence of statistically significant GMV changes of amygdala in females.

Finally, statistically significant associations between obesity and crystallized intelligence in both sex were observed in this study. Crystallized intelligence, defined as accumulated knowledge and experience reliant on memory and learning [71], showed stronger correlations with right amygdala volume changes in the obesity group. The locus coeruleus–sympathetic–adrenal medullary axis and hypothalamic–pituitary–adrenal axis, which project to the amygdala, are critical for lifelong cognitive and emotional processing [5]. Adolescent with obesity induced by high-fat diets alters the reactivity of these axes, enhancing amygdala-dependent synaptic and memory processes [72]. The right amygdala, central to negative emotions (e.g., anxiety, fear) and stress responses, may become hyperactive due to chronic metabolic stress (e.g., elevated cortisol levels) in obesity. This hyperactivity could impair prefrontal cortex resource allocation, thereby disrupting the integration of experiences and knowledge retrieval essential for crystallized intelligence [73]. Additionally, the conclusions of this study may generalize to healthy adults across other age groups, as supported by relevant research: the life-span development of resting-state functional connectivities (rsFC) in the amygdala may underlie age-related differences in emotion regulatory mechanisms. These age-related connectivity changes appear to be more pronounced in males than in females [74]. Notably, the ventrolateral amygdala-a subregion of the amygdala specialized in social perception-shows connections with social cognitive and emotional systems that follow an inverted U-shaped trajectory across the lifespan [75]. Age-specific alterations in amygdala functional connectivity indirectly facilitate the development and maintenance of crystallized intelligence [76].

However, there are some limitations to this study. Firstly, this study with the cross-sectional design cannot confirm whether these brain structural changes are an early indicator of obesity or the result of obesity. Future prospective studies are necessary to explore this issue. Secondly, the findings of this study primarily relied on structural MRI data, and future studies could complement these results with multimodal data. Finally, this study only covers young adulthood, lacking data from adolescent and older adult populations. Furthermore, while it identifies correlations, the causal mechanisms underlying these findings await experimental validation.

## Conclusion

In sum, male young adults with obesity showed significantly higher GMV of right amygdala than lean young adults, which was significantly correlated with crystallized intelligence. These findings indicated the sex difference of adverse effect of obesity might be associated with GMV of right amygdala.

## Supplementary Information

Supplementary Material 1.

## Data Availability

All data were provided by the Human Connectome Project, WU-Minn Consortium (Principal Investigators: David Van Essen and Kamil Ugurbil; 1U54MH091657) funded by the 16 NIH Institutes and Centers that support the NIH Blueprint for Neuroscience Research; and by the McDonnell Center for Systems Neuroscience at Washington University in St. Louis. The authors are grateful to the Human Connectome Project for open access to its data.
