# Supplementary material for: Sex-related difference of subcortical gray matter volume associated with crystallized intelligence in young adults with obesity
Source: Biol Sex Differ. 2026 Feb 8;17:62. doi: 10.1186/s13293-026-00850-8 (PMC13047815; doi:10.1186/s13293-026-00850-8)
Supplement: Supplementary file 1 — Supplementary Material 1. [file 13293_2026_850_MOESM1_ESM.docx]

Supplemental Materials

***1. Methods***

***1.1 Participants and sample size***

All participants were young adults between 22-35 years old and did not meet the following exclusion criteria: history of psychiatric disorder, substance abuse, neurodevelopmental disorder or damage, cardiovascular disease, severe health conditions (such as diabetes, multiple sclerosis, cerebral palsy, premature birth), or MRI contraindications (large tattoos, non-removable piercings, metal devices in the body or claustrophobia, etc.).

In this study, all participants were categorized into two groups according to their BMI (young adults with obesity: BMI > 30; lean individuals: 18 < BMI < 25)[1, 2]. Individuals with a BMI between 25 and 30 were excluded to create two separate groups of individuals with clinically significant differences in BMI. Furthermore, the R-based "MatchIt" package was adopted to match young adults with obesity and lean individuals in terms of demographic variables including sex, age, total family income and education level. Hence, the total sample in this study comprised 486 participants (243 young adults with obesity, and 243 lean individuals).

***1.2 MRI data acquisition and preprocessing***

In the HCP dataset, T1-weighted structural images were collected using a 32-channel head coin on a 3T Siemens Skyra scanner (Siemens AG, Erlanger, Germany) with the following scanning parameters: a resolution of 0.7 mm^3^ isotropic, field of view $=$ 224 mm $\times$ 240 mm, matrix size $=$ 320 $\times$ 320, repetition time $=$ 2400 ms, echo time $=$ 2.14 ms, inversion time $=$ 1000 ms, flip angle $=$ 8°, and 256 sagittal slices.

***1.3 MRI preprocessing and surface-based morphology analysis***

Data were reconstructed and preprocessed using a modified version of the FreeSurfer pipeline [3] in FreeSurfer Image Analysis Suite version 5.3 (http://surfer.nmr.mgh.harvard.edu) [4]. For details of acquisition parameters, reconstruction, and preprocessing of the HCP structural MRI data, see [5, 6]. All structural images were reviewed by a technician immediately following acquisition to ensure scans were without any significant problems (i.e., artifacts, substantial movement). For full explanation of HCP quality control, see [7]. Then, quantitative measures of CT and CSA for cortical regions defined by the Desikan atlas [8], and GMV for subcortical regions and brain stem from the ASEG parcellation plus the intracranial volume (ICV) were derived in FreeSurfer [4].

| ***Table S1.*** The clinical and neuropsychological assessments adopted in this study. | |
| --- | --- |
| Assessment | Description |
| Sleep quality | The total score across all items on the Pittsburgh Sleep Quality Index (PSQI) (Buysse et al., 1989). |
| Impulsive trait | The mean area under the curvefor Discounting of $200 and $40K.  A summary measure of Delay Discounting, the AUC discounting measure provides an index of how steeply the participant discounts a delayed reward of $200 and $40K (Myerson et al. 2001). |
| Fluid cognitive ability | The Fluid Cognition Composite score is derived by averaging the normalized scores of each of the Toolbox tests that are fluid ability measures (Flanker, Dimensional Change Card Sort, Picture Sequence Memory, List Sorting and Pattern Comparison), then deriving scale scores based on this new distribution. Higher scores indicate higher levels of functioning. |
| Crystal cognitive ability | The Crystallized Cognition Composite score is derived by averaging the normalized scores of each of the Toolbox tests that are crystallized measures (Picture Vocabulary and Reading Tests), then deriving scale scores based on this new distribution. One can interpret the Crystallized Cognition Composite as a more global assessment of individual and group verbal reasoning. Higher scores indicate higher levels of functioning. |
| General cognitive ability | The Cognitive Function Composite score is derived by averaging the normalized scores of each of the Fluid and Crystallized cognition measures, then deriving scale scores based on this new distribution. Higher scores indicate higher levels of cognitive functioning. |
| WM accuracy | Accuracy across all conditions in WM task. |
| WM RT | Average of Median Reaction Time for all conditions in WM task. |
| 2Back-WM accuracy | Accuracy across all conditions in 2-back. |
| 2Back-WM RT | Average of Median Reaction Time for all conditions in 2-back. |
| 0Back-WM accuracy | Accuracy across all conditions in 0-back. |
| 0Back-WM RT | Average of Median Reaction Time for all conditions in 0-back. |
| Aggressive behavior level | The score of ASR Aggressive Behavior (scale VI). |
| Total problems level | The total score of ASR (all questions). |
| Note. WM, working memory; RT, response time; ASR, adult self report. | |

***Figure S1.*** there were no significant "group"$\times$"sex" interaction effect on all cortical regions based on CT.


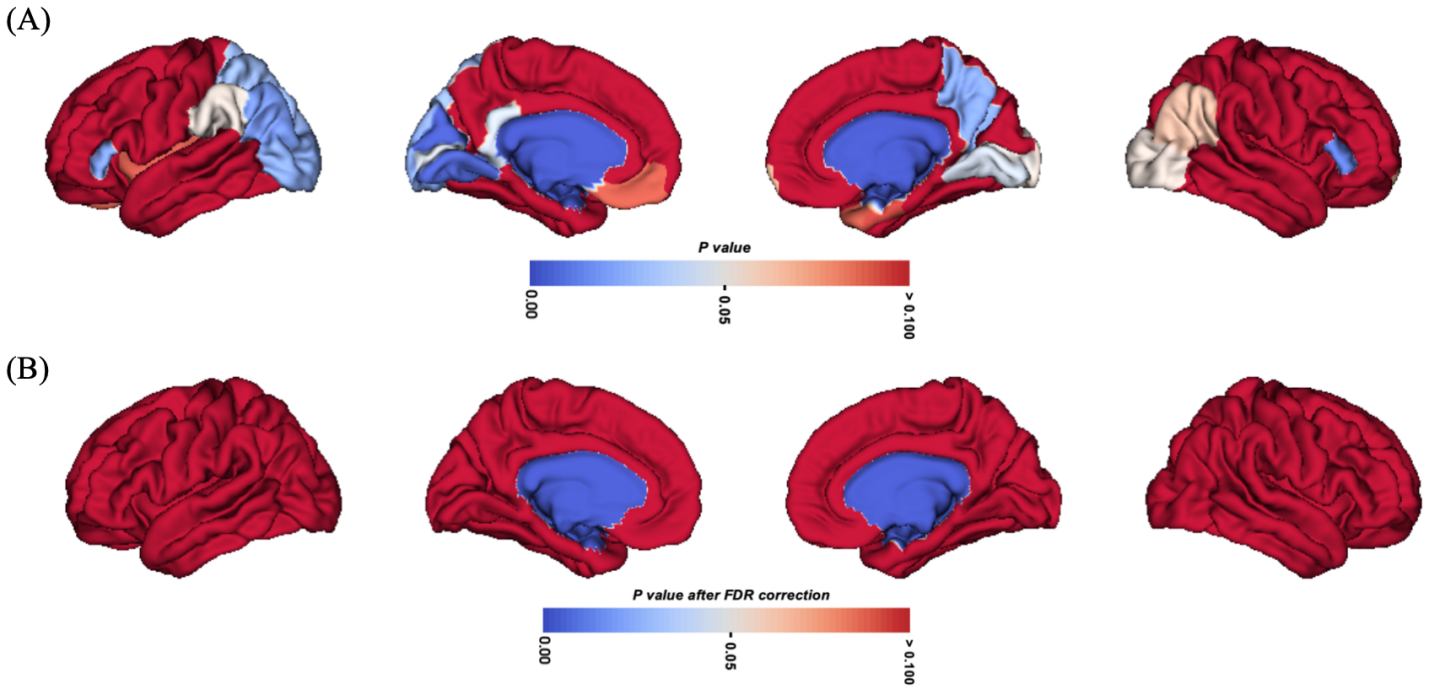


***Figure S2.*** there were no significant "group"$\times$"sex" interaction effect on all cortical regions based on CSA.


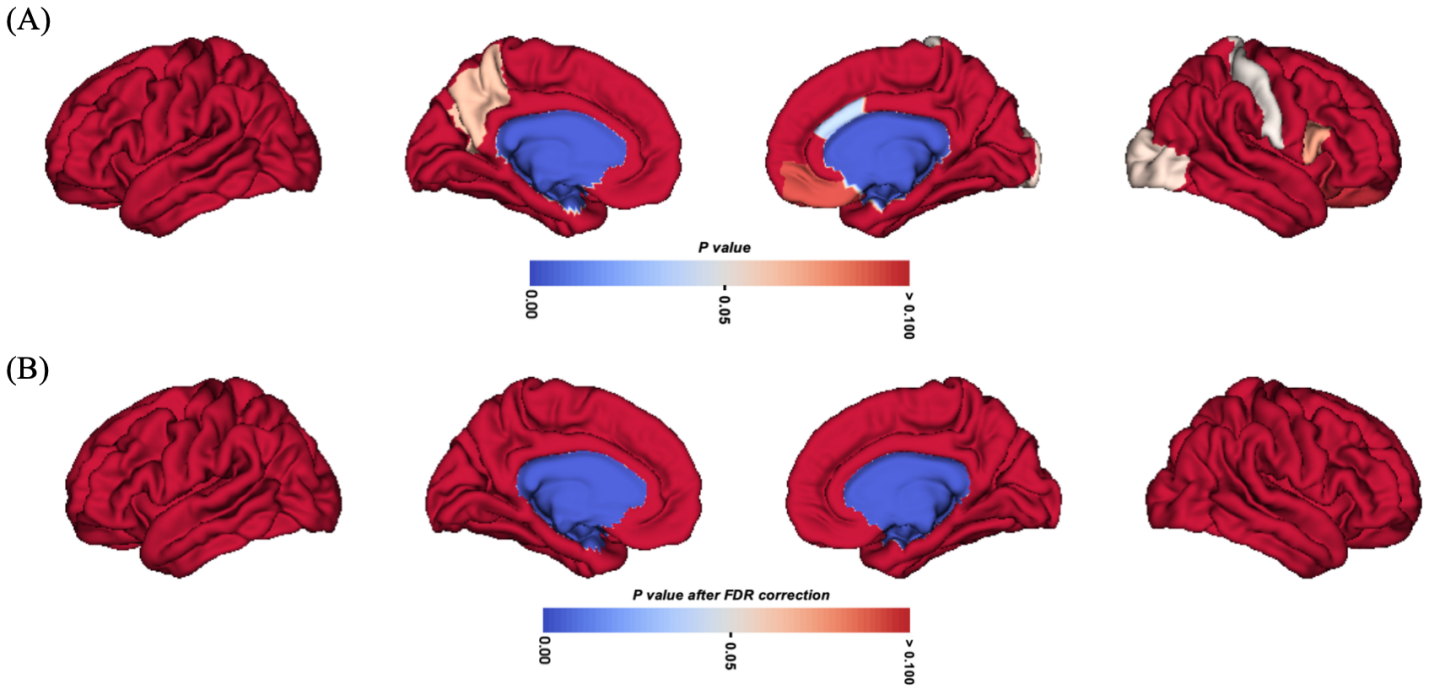


Reference:

1. *"Overweight and obesity", in Health at a Glance: Asia/Pacific 2020: Measuring*

*Progress Towards Universal Health Coverage.*

2. Xu, S., et al., *Altered structural node of default mode network mediated general cognitive ability in young adults with obesity.* Prog Neuropsychopharmacol Biol Psychiatry, 2024. **135**: p. 111132.

3. Fischl, B., et al., *Automatically parcellating the human cerebral cortex.* Cereb Cortex, 2004. **14**(1): p. 11-22.

4. Fischl, B., *FreeSurfer.* Neuroimage, 2012. **62**(2): p. 774-81.

5. Glasser, M.F., et al., *The minimal preprocessing pipelines for the Human Connectome Project.* Neuroimage, 2013. **80**: p. 105-24.

6. Van Essen, D.C., et al., *The Human Connectome Project: a data acquisition perspective.* Neuroimage, 2012. **62**(4): p. 2222-31.

7. Marcus, D.S., et al., *Human Connectome Project informatics: quality control, database services, and data visualization.* Neuroimage, 2013. **80**: p. 202-19.

8. Desikan, R.S., et al., *An automated labeling system for subdividing the human cerebral cortex on MRI scans into gyral based regions of interest.* Neuroimage, 2006. **31**(3): p. 968-80.
